# Hypertensive disorders of pregnancy and peripartum cardiomyopathy: A nationwide cohort study

**DOI:** 10.1371/journal.pone.0211857

**Published:** 2019-02-20

**Authors:** Ida Behrens, Saima Basit, Jacob A. Lykke, Mattis F. Ranthe, Jan Wohlfahrt, Henning Bundgaard, Mads Melbye, Heather A. Boyd

**Affiliations:** 1 Department of Epidemiology Research, Statens Serum Institut, Copenhagen, Denmark; 2 Department of Obstetrics, Copenhagen University Hospital (Rigshospitalet), Copenhagen, Denmark; 3 Unit for Inherited Cardiac Disease, The Heart Centre, Copenhagen University Hospital (Rigshospitalet), Copenhagen, Denmark; 4 Department of Medicine, Stanford University Medical School, Stanford, California, United States of America; University of Missouri Columbia, UNITED STATES

## Abstract

**Background:**

Peripartum cardiomyopathy (PPCM) is a serious cardiac disorder occurring late in pregnancy or early in the postpartum period. We examined associations between hypertensive disorders of pregnancy (HDP: preeclampsia and gestational hypertension) and PPCM, accounting for other pregnancy-related risk factors for PPCM.

**Methods:**

Using nationwide Danish register data, we constructed a cohort of all women with ≥1 live birth or stillbirth in Denmark between 1978 and 2012. Using log-linear binomial regression and generalized estimating equations, we estimated risk ratios (RRs) for PPCM associated with HDP of varying severity.

**Results:**

In a cohort of 1,088,063 women with 2,078,822 eligible pregnancies, 126 women developed PPCM (39 in connection with an HDP-complicated pregnancy). The risks of PPCM were significantly higher in women with HDP-complicated pregnancies than in women with normotensive pregnancies (severe preeclampsia, RR 21.2, 95% confidence interval [CI] 12.0–37.4; moderate preeclampsia, RR 10.2, 95% CI 6.18–16.9; gestational hypertension, RR 5.16, 95% CI 2.11–12.6). The RRs for moderate preeclampsia and gestational hypertension were not significantly different from one another (p = 0.18); the RR for severe preeclampsia was significantly different from the RR for moderate preeclampsia and gestational hypertension combined (p = 0.02).

**Conclusions:**

Although 70% of PPCM occurred in women with normotensive pregnancies, HDPs were associated with substantial increases in PPCM risk that depended on HDP severity. The heart’s capacity to adapt to a normal pregnancy may be exceeded in some women already susceptible to cardiac insult, contributing to PPCM. HDPs, severe preeclampsia in particular, probably represent an additional cardiac stressor during pregnancy.

## Introduction

Peripartum cardiomyopathy (PPCM) is a rare but serious cardiac disorder characterized by systolic dysfunction and symptoms of heart failure in late pregnancy or the “months following delivery” [1]. Subsequent rates of cardiac function recovery and left ventricular ejection fraction normalization vary widely (21–72% within 6–12 months of diagnosis) [2,3]; the postpartum risk of cardiovascular morbidity and mortality may be substantial [1–5]. One of the largest post-PPCM follow-up studies to date (n = 182) found that 21% of women suffered a major cardiovascular event within a year of PPCM diagnosis [5], while in another large study, 13% of women died within 6 months of a PPCM diagnosis [2].

The etiology of PPCM is not fully understood, but a link between preeclampsia and PPCM was suggested as early as the 1930’s [6]. A recent meta-analysis showed that women with PPCM were four times as likely as women without PPCM to have preeclampsia [7], and large studies have also documented associations with gestational hypertension and eclampsia [8–11]. Other suggested PPCM risk factors include multiple pregnancy, increasing parity, advanced maternal age, chronic hypertension, obesity, smoking, and genetics [7,10,12–14], most of which are also linked to hypertensive disorders of pregnancy (HDP) [15,16]. Recent advances in the understanding of PPCM pathophysiology indicate that HDP and PPCM could be linked by processes involving endothelial and cardio-toxic factors such as e.g. sFlt-1 and 16 kDa prolactin [12,17–19].

The Heart Failure Association of the European Society of Cardiology recently called for large studies to elucidate the epidemiology of PPCM and its pathogenic mechanisms [1]. Although studies have shown HDP to be a risk factor for PPCM [7–12,17], its importance relative to other risk factors is less clear. We conducted a register-based cohort study to investigate the risk of PPCM associated with hypertensive disorders of pregnancy, accounting for other PPCM risk factors.

## Methods

### Cohort

All Danish residents are assigned a unique personal identification number via which vital statistics and contacts with the health care system are registered. Using the Civil Registration System [20], the National Patient Register [21], and the Medical Birth Register [22], we identified all pregnancies in Denmark ending in live birth or stillbirth between 1978 and 2012. We excluded pregnancies of <20 weeks’ duration and all pregnancies in women registered in the National Patient Register with any cardiovascular disease (International Classification of Diseases, 8^th^ revision [ICD-8] codes 390.99–458.99, 10^th^ revision [ICD-10] codes I00.0–99.9) or diabetes (ICD-8: 249.00–250.09; ICD-10: E10.0–14.9) up to 30 days before their first delivery in the study period. Women could contribute more than one pregnancy to the study, but pregnancies occurring after a registration of cardiomyopathy (see Outcome, below), heart failure (see Outcome, below) or ischemic heart disease (ICD-8: 410.09–414.99; ICD-10: I20.0–25.9) in the National Patient Register were not considered.

### Hypertensive disorders of pregnancy (exposure)

A woman was considered to have an HDP in a given pregnancy if she had a diagnosis of gestational hypertension, preeclampsia, eclampsia, or hemolysis, elevated liver enzymes and low platelets (HELLP) syndrome registered in the National Patient Register within a given timeframe. Since by definition an HDP involves incident hypertension in a pregnant woman with onset after 20 weeks’ gestation, we considered only diagnoses registered after this point. To maximize the validity of registered HDP diagnoses, we also required that at least one diagnosis be registered in the period from 30 days before to 7 days after delivery. As registered in the National Patient Register, gestational hypertension is defined as hypertension (systolic blood pressure ≥140 mmHg or diastolic blood pressure ≥90 mmHg) without accompanying proteinuria (ICD-8 code 637.00, ICD-10 codes O13.9 or O16.9), while in moderate preeclampsia, mild/moderate hypertension is accompanied by proteinuria (ICD-8 codes 637.03, 637.09 or 637.99, ICD-10 codes O14.0 or O14.9). Severe preeclampsia fulfills the criteria for moderate preeclampsia, with the addition of one or more of the following: severe hypertension (systolic blood pressure ≥160 mmHg or diastolic blood pressure ≥110 mmHg), severe proteinuria, signs of organ failure (including the HELLP syndrome), generalized seizures (ICD-8 codes 637.04, 637.19, 762.19, 762.29, or 762.39, ICD-10 codes O14.1, O14.2, or O15.0–15.9). (See S1 Definitions in the online Supporting Information for additional details.) If more than one HDP diagnosis was registered, the pregnancy was classified according to the most severe diagnosis. For women contributing more than one pregnancy to the study, exposure status was reset for each pregnancy, such that HDP in a previous pregnancy did not contribute to HDP status in the next pregnancy; resetting exposure status between pregnancies allowed us specifically to investigate the association between a current HDP and the immediate risk of PPCM in that pregnancy.

We also classified preeclampsia based on timing of delivery, with preeclampsia requiring delivery at <34 weeks’ gestation defined as early-onset and preeclampsia with delivery ≥34 weeks defined as later-onset, regardless of registered severity.

### Peripartum cardiomyopathy and peripartum heart failure (outcomes)

PPCM was defined as cardiomyopathy registered in the National Patient Register or Causes of Death Register (ICD-8: 425.99; ICD-10: I42.0–43.8, O90.3) in the peripartum period, defined as the 6-month period beginning 1 month before delivery and ending 5 months postpartum [23,24], without registration of cardiomyopathy prior to the pregnancy. In Denmark, cardiomyopathies are diagnosed according to the European Society of Cardiology’s position paper on cardiomyopathy [25] and guidelines on heart failure [26]; since the mid-1990s, echocardiography or magnetic resonance imaging have been required to confirm a cardiomyopathy diagnosis. A PPCM diagnosis requires a left ventricular ejection fraction <45% and an absence of pre-existing (pre-pregnancy) dilated cardiomyopathy or other conditions that could produce similar structural changes in the heart (e.g. thyrotoxicosis), as per the European Society of Cardiology position paper on PPCM [1].

Since PPCM might also have been coded as heart failure, we also identified women with heart failure in the peripartum period (PPHF) (ICD-8: 427.09–427.19, 427.99, 428.99, 782.49; ICD-10: I50.0–50.9) for a separate, parallel analysis. As with the cardiomyopathies, heart failure in Denmark is diagnosed according to European Society of Cardiology guidelines [26] and requires a left ventricular ejection fraction <45%, as measured by echocardiography or magnetic resonance imaging.

### Covariates

Parity (1, 2, ≥3 live births or stillbirths), maternal age at delivery (<25, 25–29, 30–34, ≥35 years), year of delivery (5-year intervals), multiple gestation (yes/no), first-trimester smoking (yes/no) and pre-pregnancy body mass index (BMI, <25, 25–29, 30–34, ≥35), all available from the Medical Birth Register, were considered as potential confounders of the association between HDP and PPCM. We also considered the impact on our results of incident diabetes (ICD-8 codes 249.00–250.09 and ICD-10 codes E10.0–14.9 registered in the National Patient Register) and hypertension (≥2 filled prescriptions for anti-hypertensive medication [Anatomic Therapeutic Chemical codes C02, 03, 07–09] registered in the National Prescription Register [27] within 6 months in a previous non-user) that developed between pregnancies. Information on parity, maternal age, delivery year, multiple gestation, and diabetes was available for the entire study period; information on smoking, BMI, and anti-hypertensive medication use was available from 1991, 2004 and 1994, respectively.

### Statistical analyses

We used log-linear binomial regression with generalized estimating equations to estimate risk ratios (RRs) for the association between HDP and PPCM in the same pregnancy while accounting for potential within-woman correlations in women contributing more than one pregnancy. All RRs were adjusted for parity, maternal age, delivery year, and multiple gestation. We additionally adjusted for smoking and BMI in sub-cohorts of women with pregnancies in or after 1991 and 2004, respectively. Furthermore, we examined the effect of excluding pregnancies occurring after diabetes and hypertension registered after the first pregnancy in the study period, in the full cohort and a sub-cohort of women with pregnancies in or after 1995, respectively. Analyses were performed using SAS statistical software, version 9.4 (SAS Institute, Inc., Cary, N.C.).

### Ethics

Studies based solely on data from the Danish national registers do not require approval from the Danish research bioethics committees, as study participants are never contacted, and consent is not required for the use of register information. The study’s use of register data was covered by the approval extended by the Danish Data Protection Agency to all register-based studies conducted by Statens Serum Institut (approval no. 2015-57-0102).

## Results

The cohort included 1,088,063 million women with 2,078,822 eligible pregnancies in the study period, of which 76,808 (3.7%) were HDP-affected. In this cohort, 126 women were diagnosed with PPCM, 39 (31.0%) in connection with an HDP-complicated pregnancy and 87 in connection with a normotensive pregnancy. Most PPCMs were diagnosed in the last month of pregnancy or the first month postpartum, both in women with (n = 32, 82.1%) and without (n = 71, 81.6%) HDP in the affected pregnancy.

**Fig 1:**
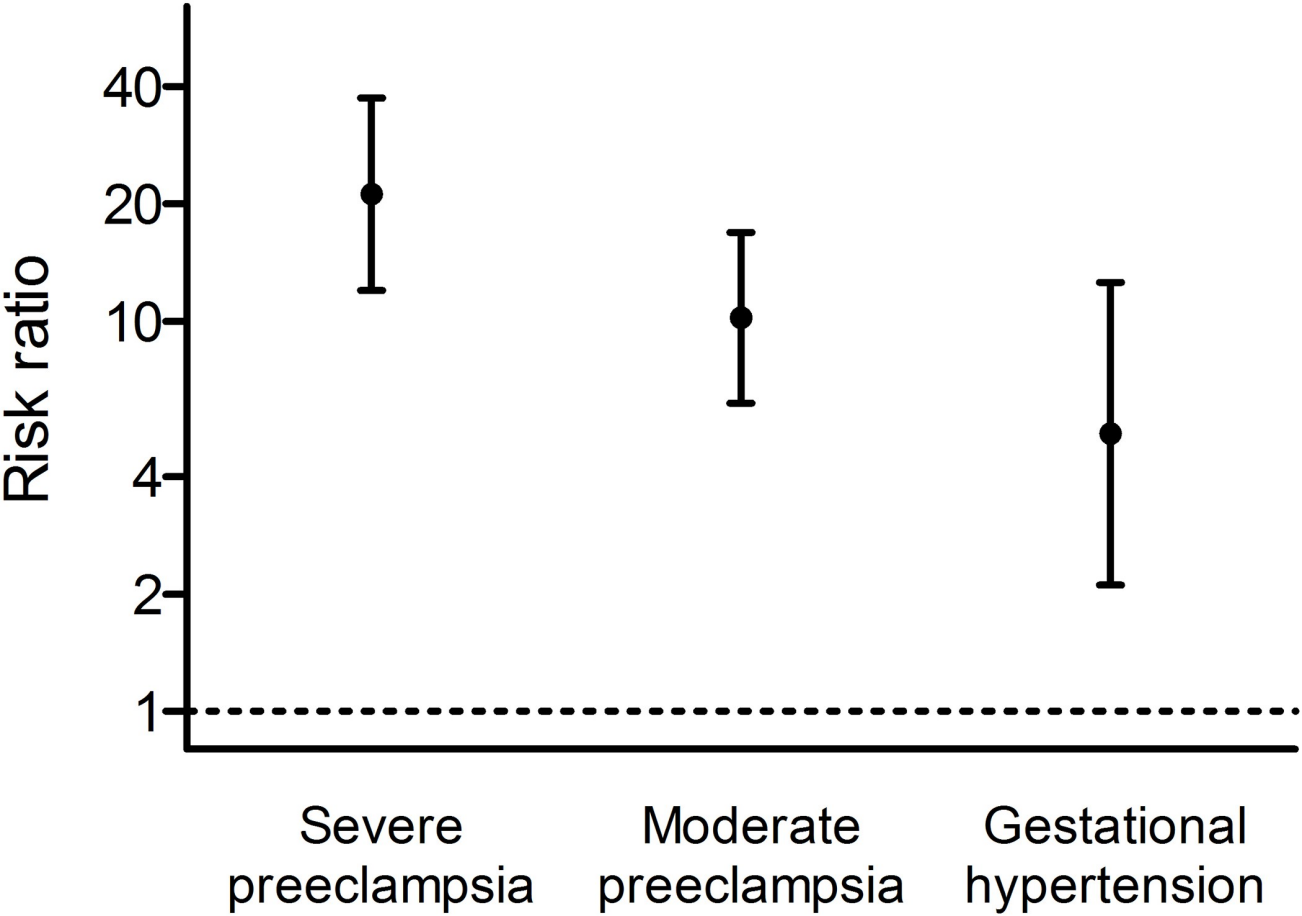
Risk ratios, with 95% confidence intervals, for the association between severe preeclampsia, moderate preeclampsia, and gestational hypertension, and peripartum cardiomyopathy in the same pregnancy, among women with one or more birth in Denmark in the period 1978–2012. Risk ratios are adjusted for parity, maternal age and calendar period at delivery, and multiple birth.

HDP was strongly and significantly associated with PPCM, with the strength of the association appearing to increase with HDP severity (Table 1, Fig 1). At least one of the RRs for severe preeclampsia, moderate preeclampsia, and gestational hypertension was significantly different from the others (p = 0.02); the RRs for moderate preeclampsia and gestational hypertension did not differ statistically from one another (p = 0.18). RR magnitudes for early and late preeclampsia did not differ (early: RR 13.2, 95% CI 4.17–41.8; late: RR 13.0, 95% CI 8.54–19.9, p = 0.99).

**Table 1:** Risk ratios for peripartum cardiomyopathy by hypertensive disorders of pregnancy and other characteristics of the pregnancy, among women with ≥1 birth, Denmark, 1978–2012.

	Pregnancies with PPCM No. (%)	Pregnancies without PPCM No. (%)	Risk ratio* (95% confidence interval)	p-value†
	N = 126	N = 2 078 696		
**Hypertensive disorder of pregnancy**				
Severe preeclampsia	15 (11.9)	13 108 (0.6)	21.2 (12.0, 37.4)	<0.0001
Moderate preeclampsia	19 (15.1)	45 018 (2.2)	10.2 (6.18, 16.9)	
Gestational hypertension	5 (4.0)	18 643 (0.9)	5.16 (2.11, 12.6)	
None	87 (69.0)	2 001 927 (96.3)	1 (ref)	
**Parity at delivery**				
1	57 (45.2)	1 087 840 (52.3)	1 (ref)	0.81
2	46 (36.5)	711 353 (34.2)	1.15 (0.75, 1.76)	
≥3	23 (18.3)	279 503 (13.4)	1.09 (0.63, 1.88)	
**Maternal age at delivery (years)**				
<25	14 (11.1)	436 081 (21.0)	1.04 (0.54, 2.00)	0.009
25–29	30 (23.8)	767 348 (36.9)	1 (ref)	
30–34	45 (35.7)	611 235 (29.4)	1.53 (0.95, 2.47)	
≥35	37 (29.4)	263 032 (12.7)	2.45 (1.44, 4.17)	
**Multiple pregnancy**				
No	116 (92.1)	2 043 844 (98.3)	1 (ref)	0.006
Yes	10 (7.9)	34 852 (1.7)	2.59 (1.32, 5.06)	
**Calendar period at delivery**				
1978–1982	2 (1.6)	276 281 (13.3)	0.24 (0.05, 1.10)	<0.0001
1983–1987	5 (4.0)	259 609 (12.5)	0.57 (0.20, 1.64)	
1988–1992	10 (7.9)	305 302 (14.7)	0.96 (0.41, 2.24)	
1993–1997	12 (9.5)	328 378 (15.8)	1 (ref)	
1998–2002	21 (16.7)	313 814 (15.1)	1.75 (0.87, 3.54)	
2003–2007	22 (17.5)	245 113 (11.8)	2.23 (1.10, 4.51)	
2008–2012	54 (42.9)	350 199 (16.8)	3.54 (1.89, 6.64)	

PPCM, peripartum cardiomyopathy.

*All risk ratios mutually adjusted for the other covariates.

†p-value for the overall association with the variable.

Maternal age >35 years and multiple gestations were associated with a higher risk of PPCM, and the PPCM risk increased over the study period (p for trend = 0.0005) (Table 1). In contrast, increasing parity was not associated with PPCM (Table 1). In a sub-cohort with smoking information, smoking was associated with an increased risk of PPCM (RR 2.42, 95% CI 1.51–3.89), but adjustment for smoking did not change the magnitude of the observed associations between HDP and PPCM (S1 Table). In a sub-cohort with BMI information, a BMI ≥35 was associated with a significantly increased risk of PPCM compared with women with BMI <25 (RR 2.42, 95% CI 1.10–5.32) (Table 2). Adjustment for BMI slightly reduced the RRs for moderate preeclampsia and gestational hypertension but did not affect our conclusions (Table 2). Likewise, excluding pregnancies following a diagnosis of diabetes had little effect on the estimates (S2 Table). When we excluded pregnancies occurring after the development of chronic hypertension, the pattern of association with HDP severity remained the same (S2 Table).

**Table 2:** Risk ratios for peripartum cardiomyopathy by hypertensive disorders of pregnancy in the same pregnancy, additionally adjusted for pre-pregnancy body mass index, among women with ≥1 birth, Denmark, 2004–2012.

	Pregnancies with PPCM No. (%)	Pregnancies without PPCM No. (%)	Risk ratio (95% confidence interval)*		
			Not adjusted for BMI	Additionally adjusted for BMI	p-value†
	N = 60	N = 493 316			
**Hypertensive disorder of pregnancy**					
Severe preeclampsia	11 (18.3)	4065 (0.8)	35.2 (17.1, 72.2)	32.5 (15.7, 67.2)	<0.0001
Moderate preeclampsia	12 (20.0)	9938 (2.0)	16.1 (8.22, 31.6)	14.3 (6.96, 29.5)	
Gestational hypertension	2 (3.3)	5785 (1.2)	4.48 (1.08, 18.6)	3.88 (0.94, 16.0)	
None	35 (58.3)	473 528 (96.0)	1 (ref)	1 (ref)	
**Pre-pregnancy body mass index (BMI)**					
<25	34 (56.7)	330 626 (67.0)	-	1 (ref)	0.046
25–29	9 (15.0)	103 880 (21.1)	-	0.72 (0.34, 1.51)	
30–34	9 (15.0)	38 854 (7.9)	-	1.67 (0.77, 3.63)	
≥35	8 (13.3)	19 956 (4.0)	-	2.42 (1.10, 5.32)	

BMI, body mass index. PPCM, peripartum cardiomyopathy.

*All risk ratios are adjusted for parity, maternal age at delivery, multiple pregnancies, and calendar period at delivery. Information on pre-pregnancy BMI was available from 2004. Consequently, the risk ratios presented in the table are based on the subset of pregnancies in the period 2004–2012 where the woman’s pre-pregnancy BMI was known (493,376/527,571 pregnancies, 93.5%). When pregnancies with missing pre-pregnancy BMI (n = 33,942, 6.4%) were also included in the analyses, the results changed very little: the risk ratios for severe preeclampsia, moderate preeclampsia and gestational hypertension were 35.7 (95% CI 18.0, 70.7), 13.9 (95% CI 6.84, 28.3) and 5.69 (95% CI 1.73, 18.8), respectively, while the risk ratios for BMI 25–29, 30–34, ≥35 and missing were 0.71 (95% CI 0.38, 1.50), 1.65 (95% CI 0.76, 3.58), 2.37 (95% CI 1.08, 5.22) and 1.92 (95% CI 0.80, 4.61), respectively.

†p-value for the overall association with the variable, in the model with additional adjustment for BMI.

During the study period, 89 women were diagnosed with PPHF without an accompanying PPCM diagnosis. Among the women with an HDP-complicated pregnancy and PPHF (n = 20), 90% were diagnosed with PPHF within a month of delivery, whereas only 68% of the women with a normotensive pregnancy and PPHF (n = 69) received their PPHF diagnosis in the same time window. Similar to PPCM, women with HDP, particularly those with severe preeclampsia, were at increased risk of PPHF compared with women with normotensive pregnancies (Table 3).

**Table 3:** Risk ratios for peripartum heart failure by hypertensive disorders of pregnancy and other characteristics of the pregnancy, among women with ≥1 pregnancy, Denmark, 1978–2012.

	Pregnancies with PPCM No. (%)	Pregnancies without PPCM No. (%)	Risk ratio* (95% confidence interval)	p-value†
	N = 126	N = 2 078 696		
**Hypertensive disorder of pregnancy**				
Severe preeclampsia	12 (13.5)	13 108 (0.6)	27.5 (14.5, 51.9)	<0.001
Moderate preeclampsia	6 (6.7)	45 018 (2.2)	3.87 (1.69, 8.87)	
Gestational hypertension	2 (2.2)	18 643 (0.9)	3.05 (0.75, 12.5)	
None	69 (77.5)	2 001 927 (96.3)	1 (ref)	
**Parity at delivery**				
1	45 (50.6)	1 087 840 (52.3)	1 (ref)	0.44
2	31 (34.8)	711 353 (34.2)	1.32 (0.80, 2.20)	
≥3	13 (14.6)	279 503 (13.4)	1.43 (0.74, 2.74)	
**Maternal age at delivery (years)**				
<25	17 (19.1)	436 081 (21.0)	1.05 (0.55, 1.99)	0.41
25–29	27 (30.3)	767 348 (36.9)	1 (ref)	
30–34	32 (36.0)	611 235 (29.4)	1.48 (0.90, 2.43)	
≥35	13 (14.6)	263 032 (12.7)	1.32 (0.69, 2.54)	
**Multiple pregnancy**				
No	83 (93.3)	2 043 844 (98.3)	1 (ref)	0.01
Yes	6 (6.7)	34 852 (1.7)	3.06 (1.31, 7.16)	
**Calendar period at delivery**				
1978–1982	17 (19.1)	276 281 (13.3)	2.91 (1.22, 6.93)	0.09
1983–1987	9 (10.1)	259 609 (12.5)	1.42 (0.55, 3.63)	
1988–1992	18 (20.2)	305 302 (14.7)	2.29 (1.03, 5.14)	
1993–1997	9 (10.1)	328 378 (15.8)	1 (ref)	
1998–2002	9 (10.1)	313 814 (15.1)	1.02 (0.41, 2.57)	
2003–2007	12 (13.5)	245 113 (11.8)	1.68 (0.71, 3.95)	
2008–2012	15 (16.9)	350 199 (16.8)	1.40 (0.62, 3.16)	

PPHF, peripartum heart failure.

*All risk ratios mutually adjusted for the other covariates.

†p-value for the overall association with the variable.

## Discussion

### Main findings and interpretation

Preeclampsia has long been considered a risk factor for PPCM, and previously published data suggested that PPCM risk might depend on HDP severity [8–11]. In a nationwide study of >2 million pregnancies, we showed that PPCM risk in women with HDP was dramatically increased (5–21 times) compared with the risk in women with normotensive pregnancies, and demonstrated that the risk associated with severe preeclampsia was greater than that associated with moderate preeclampsia and gestational hypertension.

A mechanistic link between HDP and PPCM is biologically plausible. An angiogenic imbalance, with increased levels of anti-angiogenic factors (e.g. sFlt-1), is believed to play a role in the pathophysiology of HDP [28], and sFlt-1 and anti-angiogenic prolactin cleavage products may also be involved in the pathophysiology of PPCM [17–19]. The altered hemodynamics of HDP also increase cardiac stress during pregnancy. Up to 80% of preeclamptic women exhibit adaptive cardiac changes such as myocardial remodeling, and up to 50% have left ventricular global diastolic dysfunction; furthermore, 20% of women with severe forms of preeclampsia experience even greater cardiac dysfunction (left ventricular hypertrophy and radial systolic dysfunction) [29]. Why only some women with HDP develop PPCM can perhaps be explained by a “two-hit” hypothesis, whereby the anti-angiogenic state and/or cardiac changes common in HDP push women already susceptible to cardiac insult (due to e.g. reduced cardiac proangiogenic defenses, myocarditis, genetic variants, viral infection [14,17,19]) over some homeostatic threshold. Our finding that severe preeclampsia was most strongly associated with PPCM fits well with this hypothesis, since women with severe preeclampsia often have high levels of anti-angiogenic factors [30] and significant cardiac maladaptation [31].

Despite the magnitude of the associations between HDP and PPCM, most PPCM (69%) occurred in apparently healthy women with normotensive pregnancies. The hemodynamic load of a normal pregnancy may also exceed the heart’s adaptive capacity in a certain proportion of women, a suggestion supported by the recent finding that 18–28% of apparently healthy nulliparous women had left ventricular diastolic chamber dysfunction and/or impaired myocardial relaxation at term [32]. Alternatively, pregnancy may simply trigger cardiomyopathy in women already predisposed to cardiac pathology. The distribution of truncating variants in genes previously associated with dilated cardiomyopathy (e.g. the gene for the sarcomere protein titin) is similar for women with PPCM and persons with idiopathic dilated cardiomyopathy [14], suggesting that in women already predisposed to dilated cardiomyopathy, the stress of pregnancy may simply hasten the process, resulting in PPCM.

As observed in other studies, higher maternal age [8,11] and multiple pregnancy [8,11] were independently associated with the risk of PPCM. Smoking was also strongly associated with PPCM, as seen in other studies [12,13]. We also found a strong association between high BMI and PPCM risk, which is at odds with the prevalent opinion about the importance of obesity in the etiology of PPCM [8,13,19]. Interestingly, adjustment for BMI did not meaningfully affect the associations between HDP and PPCM, suggesting that the observed associations cannot be explained by obesity. Other studies have observed associations between both chronic hypertension and diabetes, and PPCM [8,10,11], but our results were robust to the exclusion of pregnancies following the development of diabetes and hypertension. There were too few adverse outcomes among women with PPCM (e.g. only one death among women with both an HDP and PPCM, and 12 deaths in women with PPCM and no HDP) to allow us to assess whether there were HDP-related differences in PPCM outcomes.

### Strengths and potential limitations

By basing our study on all women in Denmark giving birth in a given period, we minimized the possibility of selection bias. Using information from the Civil Registration System minimized loss to follow-up, and the use of prospectively collected diagnosis information from Denmark’s health registers ensured freedom from recall bias.

The validity of any register-based study depends on the correctness and completeness with which diagnoses of interest are registered. Although the sensitivity of the National Patient Register for HDP and heart failure ranges from low to moderate (10% for gestational hypertension, 69% for preeclampsia overall and 29% for heart failure), the specificity for both conditions is very high (≥99%) [33,34]. Consequently, non-differential bias due to misclassification of HDP or heart failure status is likely to have been negligible.

Preeclampsia definitions have evolved considerably in the 40 years since the beginning of our study period. Of particular relevance to our study, over the past few years, the American College of Obstetrics and Gynecology, the Royal Society of Obstetrics and Gynecology, and the International Society for the Study of Hypertension in Pregnancy [ISSHP], the professional societies whose definitions and guidelines are accepted internationally, have moved away from making clinical distinctions between mild or moderate preeclampsia and severe preeclampsia. The ISSHP’s 2018 guidelines no longer include the term “mild” or “moderate preeclampsia” [35]. However, for the majority of the study period, these terms were routinely used. Both the ICD-8 (used in Denmark until 1996) and ICD-10 (in use since 1996) systems code preeclampsia diagnoses as “moderate” or “severe”, and physicians continue to code preeclampsia using ICD-10 codes, as the ICD system has not yet caught up with the guidelines. Consequently, although we use outdated terms (defined carefully in the Methods and online Supporting Information), our findings remain clinically relevant and show a clear pattern with increasing disease severity. For comparison, we also provided results based on timing of preeclampsia onset (any preeclampsia, categorized by timing of delivery), as is now preferred.

Cardiomyopathies registered in the National Patient Register have also been validated against medical records; in a large validation study, 90% of all registered cardiomyopathy diagnoses, and 75% of registered dilated cardiomyopathy diagnoses (the form of cardiomyopathy typically seen in PPCM), could be confirmed [36]. Danish cardiologists follow European Society for Cardiology guidelines when diagnosing PPCM and the other cardiomyopathies, and assignment of a cardiomyopathy diagnosis is preceded by a thorough clinical work-up that since the mid-1990s has included echocardiography. To further ensure that our results were based on valid PPCM diagnoses, we excluded from the study women with any form of cardiovascular disease up to one month before their first delivery in the study period. Consequently, one would expect the specificity of PPCM diagnoses included in the study to be excellent. In fact, the prevalence of PPCM in our study (ca. 1:16,500 deliveries) was lower than prevalences reported in many other studies [37], suggesting that our definition of PPCM was conservative. Our study also stretched over a 35-year period and PPCM was likely underdiagnosed before the advent of echocardiography, which would tend to lower the PPCM prevalence averaged over the whole study period. (Indeed, the prevalence in the period 2008–2012 was ca. 1:6,500 deliveries.) In addition, we set the upper limit of the peripartum period at five months postpartum, whereas other groups studied a longer period; however, our previous work has shown that very few additional cases of cardiomyopathy were identified between 5 months and 5 years postpartum [38]. Since the timing of PPCM diagnoses was similar for women with and without HDP, surveillance bias (due to increased medical surveillance of women with HDP) is also unlikely to explain the observed association between HDP and PPCM. Finally, since differences in access to health care among immigrant and non-immigrant women in Denmark are modest [39,40], and PPCM is such a severe condition that all cases will come to medical attention, surveillance bias due to differential access to health care for immigrants is unlikely.

Since PPCM manifests as idiopathic heart failure in the peripartum period, we expected that a certain proportion of PPCM might be coded as PPHF, particularly early in the study period (before the advent of echocardiography). We did indeed observe strong time trends, with higher incidences of PPCM later in the study period and of PPHF in earlier years. For most affected women, PPCM and PPHF were diagnosed in either the last month of pregnancy or the first month after delivery, suggesting a great deal of overlap in the conditions, and not unexpectedly, HDP, severe preeclampsia in particular, was also associated with large increases in PPHF risk.

## Conclusion

Although the majority of PPCM occurred in women with normotensive pregnancies, HDP were associated with markedly increased risks of PPCM that increased with HDP severity. The associations were robust to adjustment for other risk factors for PPCM. The heart’s capacity to adapt to a normal pregnancy may be exceeded in some women already susceptible to cardiac insult, contributing to PPCM. HDP, severe preeclampsia in particular, probably represents an additional cardiac stressor during pregnancy.

## Supporting information

S1 Definitions Definitions underlying the ICD codes used to code preeclampsia and gestational hypertension in the National Patient Register. (DOCX)

S1 Table Risk ratios for peripartum cardiomyopathy by hypertensive disorders of pregnancy, additionally adjusted for smoking, among women with ≥1 pregnancy, Denmark, 1991–2012. (DOCX)

S2 Table Risk ratios for peripartum cardiomyopathy by hypertensive disorders of pregnancy, excluding pregnancies following a) a diagnosis of diabetes mellitus or b) initiation of anti-hypertensive therapy, among women with ≥1 pregnancy, Denmark.
(DOCX)

## References

[1] Sliwa K, Hilfiker-Kleiner D, Petrie MC, Mebazaa A, Pieske B, Buchmann E, et al Current state of knowledge on aetiology, diagnosis, management, and therapy of peripartum cardiomyopathy: a position statement from the Heart Failure Association of the European Society of Cardiology Working Group on peripartum cardiomyopathy. Eur J Heart Fail. 2010;12: 767–778. 10.1093/eurjhf/hfq120 20675664

[2] Blauwet LA, Libhaber E, Forster O, Tibazarwa K, Mebazaa A, Hilfiker-Kleiner D, et al Predictors of outcome in 176 South African patients with peripartum cardiomyopathy. Heart. 2013;99: 308–313. 10.1136/heartjnl-2012-302760 23118348

[3] McNamara DM, Elkayam U, Alharethi R, Damp J, Hsich E, Ewald G, et al Clinical outcomes for peripartum cardiomyopathy in North America: results of the IPAC study (Investigations of Pregnancy-Associated Cardiomyopathy). J Am Coll Cardiol. 2015;66: 905–914. 10.1016/j.jacc.2015.06.1309 26293760 PMC5645077

[4] Sliwa K, Forster O, Tibazarwa K, Libhaber E, Becker A, Yip A, et al Long-term outcome of peripartum cardiomyopathy in a population with high seropositivity for Human Immunodeficiency Virus. Int J Cardiol. 2011;147: 202–208. 10.1016/j.ijcard.2009.08.022 19751951

[5] Goland S, Modi K, Bitar F, Janmohamed M, Mirocha JM, Czer LS, et al Clinical profile and predictors of complications in peripartum cardiomyopathy. J Card Fail. 2009;15: 645–650. 10.1016/j.cardfail.2009.03.008 19786252

[6] Hull E, Hidden E. Postpartal heart Failure. *Southern Med J*. 1938;31:265–270.

[7] Bello N, Rendon ISH, Arany Z. The relationship between pre-eclampsia and peripartum cardiomyopathy: a systematic review and meta-analysis. J Am Coll Cardiol. 2013;62: 1715–1723. 10.1016/j.jacc.2013.08.717 24013055 PMC3931606

[8] Kao DP, Hsich E, Lindenfeld J. Characteristics, adverse events, and racial differences among delivering mothers with peripartum cardiomyopathy. JACC Heart Fail. 2013;1: 409–416. 10.1016/j.jchf.2013.04.011 24163791 PMC3806506

[9] Mielniczuk LM, Williams K, Davis DR, Tang AS, Lemery R, Green MS, et al Frequency of peripartum cardiomyopathy. Am J Cardiol. 2006;97: 1765–1768. 10.1016/j.amjcard.2006.01.039 16765131

[10] Kolte D, Khera S, Aronow WS, Palaniswamy C, Mujib M, Ahn C, et al Temporal trends in incidence and outcomes of peripartum cardiomyopathy in the United States: A nationwide population-based study. J Am Heart Assoc. 2014;3: 1–13. 10.1161/JAHA.114.001056 PMC4309108 24901108

[11] Gunderson EP, Croen LA, Chiang V, Yoshida CK, Walton D, Go AS. Epidemiology of peripartum cardiomyopathy: incidence, predictors and outcomes. Obstet Gynecol. 2011;118: 583–591. 10.1097/AOG.0b013e318229e6de 21860287

[12] Haghikia A, Podewski E, Libhaber E, Labidi S, Fischer D, Roentgen P, et al Phenotyping and outcome on contemporary management in a German cohort of patients with peripartum cardiomyopathy. Basic Res Cardiol. 2013;108: 366 10.1007/s00395-013-0366-9 23812247 PMC3709080

[13] Blauwet LA, Cooper LT. Diagnosis and management of peripartum cardiomyopathy. Heart. 2011;97: 1970–1981. 10.1136/heartjnl-2011-300349 22058286

[14] Ware JS, Li J, Mazaika E, Yasso CM, DeSouza T, Cappola TP, et al Shared genetic predisposition in peripartum and dilated cardiomyopathies. N Engl J Med. 2016;374: 233–241. 10.1056/NEJMoa1505517 26735901 PMC4797319

[15] Duckitt K, Harrington D. Risk factors for pre-eclampsia at antenatal booking: systematic review of controlled studies. BMJ. 2005;330: 565 10.1136/bmj.38380.674340.E0 15743856 PMC554027

[16] England L, Zhang J. Smoking and risk of preeclampsia: a systematic review. Front Biosci. 2007;12: 2471–2483. 17127256 10.2741/2248

[17] Patten IS, Rana S, Shahul S, Rowe GC, Jang C, Liu L, et al Cardiac angiogenic imbalance leads to peripartum cardiomyopathy. Nature. 2012;485: 333–338. 10.1038/nature11040 22596155 PMC3356917

[18] Hilfiker-Kleiner D, Kaminski K, Podewski E, Bonda T, Schaefer A, Sliwa K, et al A cathepsin D-cleaved 16 kDa form of prolactin mediates postpartum cardiomyopathy. Cell. 2007;128: 589–600. 10.1016/j.cell.2006.12.036 17289576

[19] Arany Z, Elkayam U. Peripartum cardiomyopathy. *Circulation*. 2016;133:1397–1409. 10.1161/CIRCULATIONAHA.115.020491 27045128

[20] Schmidt M, Pedersen L, Sørensen HT. The Danish Civil Registration System as a tool in epidemiology. Eur J Epidemiol. 2014;29: 541–549. 10.1007/s10654-014-9930-3 24965263

[21] Lynge E, Sandegaard JL, Rebolj M. The Danish National Patient Register. Scand J Public Health. 2011;39: 30–33. 10.1177/1403494811401482 21775347

[22] Knudsen LB, Olsen J. The Danish Medical Birth Registry. Dan Med Bull. 1998;45: 320–323. 9675544

[23] Elkayam U, Akhter MW, Singh H, Khan S, Bitar F, Hameed A, et al Pregnancy associated cardiomyopathy. Clinical characteristics and a comparison between early and late presentations. Circulation. 2005;111: 2050–2055. 10.1161/01.CIR.0000162478.36652.7E 15851613

[24] European Society of Gynecology (ESG); Association for European Paediatric Cardiology (AEPC); German Society for Gender Medicine (DGesGM); Regitz-Zagrosek V, Blomstrom Lundqvist C, Borghi C, et al ESC Guidelines on the management of cardiovascular diseases during pregnancy: the Task Force on the Management of Cardiovascular Diseases during Pregnancy of the European Society of Cardiology (ESC). Eur Heart J. 2011;32: 3147–97. 10.1093/eurheartj/ehr218 21873418

[25] Elliott P, Andersson B, Arbustini E, Bilinska Z, Cecchi F, Charron P, et al Classification of the cardiomyopathies: a position statement from the European Society of Cardiology Working Group on myocardial and pericardial diseases. Eur Heart J. 2008;29: 270–276. 10.1093/eurheartj/ehm342 17916581

[26] Ponikowski P, Voors AA, Anker SD, Bueno H, Cleland JG, Coats AJ, et al 2016 ESC guidelines for the diagnosis and treatment of acute and chronic heart failure. Eur Heart J. 2016;37: 2129–2200. 10.1093/eurheartj/ehw128 27206819

[27] Kildemoes HW, Sørensen HT, Hallas J. The Danish National Prescription Registry. Scand J Public Health. 2011;39: 38–41. 10.1177/1403494810394717 21775349

[28] Powe CE, Levine RJ, Karumanchi SA. Preeclampsia, a disease of the maternal endothelium: the role of antiangiogenic factors and implications for later cardiovascular disease. Circulation. 2011;123: 2856–2869. 10.1161/CIRCULATIONAHA.109.853127 21690502 PMC3148781

[29] Melchiorre K, Sharma R, Thilaganathan B. Cardiovascular implications in preeclampsia: an overview. Circulation. 2014;130: 703–714. 10.1161/CIRCULATIONAHA.113.003664 25135127

[30] Levine RJ, Maynard SE, Qian C, Lim KH, England LJ, Yu KF, et al Circulating angiogenic factors and the risk of preeclampsia. N Engl J Med. 2004;350: 672–683. 10.1056/NEJMoa031884 14764923

[31] Melchiorre K, Sutherland GR, Watt-Coote I, Liberati M, Thilaganathan B. Severe myocardial impairment and chamber dysfunction in preterm preeclampsia. Hypertens Pregnancy. 2012;31: 454–471. 10.3109/10641955.2012.697951 23030711

[32] Melchiorre K, Sharma R, Khalil A, Thilaganathan B. Maternal cardiovascular function in normal pregnancy: Evidence of maladaptation to chronic volume overload. Hypertension. 2016;67: 754–762. 10.1161/HYPERTENSIONAHA.115.06667 26962206

[33] Kümler T, Gislason GH, Kirk V, Bay M, Nielsen OW, Køber L, et al Accuracy of a heart failure diagnosis in administrative registers. Eur J Heart Fail. 2008;10: 658–660. 10.1016/j.ejheart.2008.05.006 18539522

[34] Klemmensen AK, Olsen SF, Osterdal ML, Tabor A. Validity of preeclampsia-related diagnoses recorded in a national hospital registry and in a postpartum interview of the women. Am J Epidemiol. 2007;166: 117–124. 10.1093/aje/kwm139 17556761

[35] Brown MA, Magee LA, Kenny LC, Karumanchi SA, McCarthy FP, Saito S, et al The hypertensive disorders of pregnancy: ISSHP classification, diagnosis and management recommendations for international practice. Preg Hypertens. 2018;13: 291–310. 10.1016/j.preghy.2018.05.004 29803330

[36] Sundbøll J, Adelborg K, Munch T, Frøslev T, Sørensen HT, Bøtker HE, et al Positive predictive value of cardiovascular diagnoses in the Danish National Patient Registry: a validation study. BMJ Open. 2016;6: e012832 10.1136/bmjopen-2016-012832 27864249 PMC5129042

[37] Ersbøll AS, Damm P, Gustafsson F, Vejlstrup NG, Johansen M. Peripartum cardiomyopathy: a systematic literature review. Acta Obstet Gynecol Scand. 2016;95: 1205–1219. 10.1111/aogs.13005 27545093

[38] Behrens I, Basit S, Lykke JA, Ranthe MF, Melbye M, Bundgaard H, et al Association between hypertensive disorders of pregnancy and later risk of cardiomyopathy. JAMA. 2016;315: 1026–1033. 10.1001/jama.2016.1869 26954411

[39] Dyhr L, Andersen JS, Engholm G. The pattern of contact with general practice and casualty departments of immigrants and non-immigrants in Copenhagen, Denmark. Dan Med Bull. 2007;54: 226–229. 17850728

[40] Hansen AR, Kjøller M. Report: Health among ethnic minorities Results from the 2005 Health and Sickness Survey (SUSY-2005). National Institute of Public Health, University of Southern Denmark. 1.2005.36. [Danish]

