# Supplementary material for: Hypertensive disorders of pregnancy and peripartum cardiomyopathy: A nationwide cohort study
Source: PLoS One. 2019 Feb 20;14(2):e0211857. doi: 10.1371/journal.pone.0211857 (PMC6382119; doi:10.1371/journal.pone.0211857)
Supplement: S1 Definitions — (DOCX) [file pone.0211857.s001.docx]

# **S1 Definitions. Definitions underlying the ICD codes used to code preeclampsia and gestational hypertension in the National Patient Register**

The diagnosis of HDPs and their coding in the National Patient Register follow guidelines set out by the Danish Society for Obstetrics and Gynecology (DSOG), which have evolved over time. The DSOG guidelines in place at the end of the study period were consistent with the National Institute for Health and Clinical Excellence (NICE)/Royal College of Obstetrics and Gynaecology (RCOG) (2010) guidelines^1^ and define hypertensive disorders of pregnancy as follows (online, in Danish^2^):

Moderate preeclampsia:

Blood pressure: systolic ≥140 mmHg or diastolic ≥90 mmHg, accompanied by

Proteinuria: >300mg/24 hours or ≥1+ urine dipstick

Onset after gestational week 20

ICD-8 codes 637.03, 637.09 or 637.99; ICD-10 codes O14.0 or O14.9

Severe preeclampsia:

Fulfills the criteria for moderate preeclampsia, with the addition of either blood pressure in excess of an even greater threshold or symptoms/laboratory findings of organ involvement:

Blood pressure:

- Systolic blood pressure >160 mmHg or diastolic blood pressure ≥110 mmHg

Symptoms:

- CNS: headache, vision abnormalities, seizures (eclampsia)
- Circulatory: dyspnea, chest pressure (pulmonary edema)
- Liver: epigastric pain, vomiting

Laboratory findings:

- Liver: Elevated liver enzymes (alanine aminotransferase/aspartate aminotransferase >70 U/L), elevated serum bilirubin
- Kidney: Oliguria (<400 mL/24 hours), severe proteinuria (>3 g/24 hours), serum uric acid >0.45 mmol/L, serum creatinine >110 µmol/L = >1.24 mg/dL
- Coagulation: platelet count <100 x 10^9^/L, disseminated intravascular coagulation (activated partial thromboplastin time >1.5 x baseline value, antithrombin <70 U/dL), hemolysis (lactate dehydrogenase >600 U/L and/or haptoglobin <30 mg/dL = < 3 µmol/L)

The DSOG defines HELLP syndrome as hemolysis (as above), elevated liver enzymes (alanine aminotransferase/aspartate aminotransferase >100U/L), and low platelets (<100 x 10^9^/L). We included eclampsia and HELLP (hemolysis, elevated liver enzymes and low platelets) syndrome diagnoses under severe preeclampsia because these conditions are rare in Denmark and there were too few affected women to allow for separate groups.

ICD-8 codes 637.04, 637.19, 762.19, 762.29, or 762.39; ICD-10 codes O14.1, O14.2, or O15.0-15.9

Gestational hypertension:

Blood pressure: systolic ≥140 or diastolic ≥90 mmHg in the absence of proteinuria

Onset after 20 weeks gestation

ICD-8 codes 637.00; ICD-10 codes O13 or O16

1. National Collaborating Centre for Women’s and Children’s Health Commissioned by the National Institute for Health and Clinical Excellence. Hypertension in Pregnancy: The Management of Hypertensive Disorders During Pregnancy. London: RCOG Press; 2010.
2. Dansk Selskab for Obstetrik og Gynækologi. Hypertension og præeklampsi. DSOG Guideline. 2012;1–32. [Danish] <http://gynobsguideline.dk/sandbjerg/120403%20PIH%202012%20final.pdf>
