# Supplementary material for: Hypertensive disorders of pregnancy and peripartum cardiomyopathy: A nationwide cohort study
Source: PLoS One. 2019 Feb 20;14(2):e0211857. doi: 10.1371/journal.pone.0211857 (PMC6382119; doi:10.1371/journal.pone.0211857)
Supplement: S1 Table — (DOCX) [file pone.0211857.s002.docx]

**S1 Table. Risk ratios for peripartum cardiomyopathy by hypertensive disorders of pregnancy, additionally adjusted for smoking, among women with ≥1 pregnancy, Denmark, 1991-2012.**

|  | No. pregnancies with PPCM  N=96 | No. pregnancies without PPCM  N=1 302 504 | Risk ratio (95% confidence interval)* | |
| --- | --- | --- | --- | --- |
|  |  |  | Not adjusted for smoking | Additionally adjusted for smoking |
| HDP status |  |  |  |  |
| Severe preeclampsia | 14 | 9088 | 25.0 (13.7, 45.8) | 26.5 (14.4, 48.7) |
| Moderate preeclampsia | 15 | 25 838 | 10.7 (6.08, 18.7) | 11.0 (6.27, 19.3) |
| Gestational hypertension | 3 | 11 705 | 4.12 (1.30, 13.1) | 4.28 (1.35, 13.6) |
| Normotensive pregnancy | 64 | 1 255 873 | 1 (ref) | 1 (ref) |
| Smoking |  |  |  |  |
| Yes | 29 | 277 215 | - | 2.42 (1.51, 3.89) |
| No | 67 | 1 025 289 | - | 1 (ref) |

HDP, hypertensive disorders of pregnancy. PPCM, peripartum cardiomyopathy.

*All risk ratios are adjusted for parity, maternal age at delivery, multiple pregnancies, and calendar period at delivery. The risk ratios additionally adjusted for smoking are based on the subset of pregnancies in the period 1991-2012 where the woman’s smoking status in the first trimester (as reported at the first pregnancy visit) was known (1,302,600/1,360,351 pregnancies, 95.8%).

When pregnancies with missing smoking status (n=57,751, 4.2%) were included in the analyses, the risk ratios for severe preeclampsia, moderate preeclampsia and gestational hypertension were 22.5 (95% CI 12.5, 40.5), 10.9 (95% CI 6.40, 18.5) and 4.88 (95% CI 1.79, 13.3), respectively, while the risk ratios for smokers and women missing smoking status were 2.40 (95% CI 1.50, 3.83) and 5.10 (2.89, 8.98), respectively.

Assuming that all women with missing smoking status were smokers produced the following results: the risk ratios for severe preeclampsia, moderate preeclampsia and gestational hypertension were 24.0 (95% CI 13.5, 42.7), 11.0 (95% CI 6.48, 18.8) and 4.93 (95% CI 1.81, 13.5), respectively, while the risk ratio for smoking was 2.97 (95% CI 1.98, 4.46).

Assuming that all women with missing smoking status were non-smokers produced the following results: the risk ratios for severe preeclampsia, moderate preeclampsia and gestational hypertension were 24.3 (95% CI 13.6, 43.6), 10.9 (95% CI 6.40, 18.6) and 4.84 (95% CI 1.77, 13.2), respectively, while the risk ratio for smoking was 1.99 (95% CI 1.26, 3.15).
