# Supplementary material for: Hypertensive disorders of pregnancy and peripartum cardiomyopathy: A nationwide cohort study
Source: PLoS One. 2019 Feb 20;14(2):e0211857. doi: 10.1371/journal.pone.0211857 (PMC6382119; doi:10.1371/journal.pone.0211857)
Supplement: S2 Table — Risk ratios for peripartum cardiomyopathy by hypertensive disorders of pregnancy, excluding pregnancies following a) a diagnosis of diabetes mellitus or b) initiation of anti-hypertensive therapy, among women with ≥1 pregnancy, Denmark. (DOCX) [file pone.0211857.s003.docx]

**S2 Table. Risk ratios for peripartum cardiomyopathy by hypertensive disorders of pregnancy, excluding pregnancies following a) a diagnosis of diabetes mellitus or b) initiation of anti-hypertensive therapy, among women with ≥1 pregnancy, Denmark.**

|  | Excluding pregnancies following diabetes diagnosis (1978-2012) | | | | Excluding pregnancies following initiation of anti-hypertensive therapy (1995-2012) | | | |
| --- | --- | --- | --- | --- | --- | --- | --- | --- |
| HDP status | No. pregnancies with PPCM (N=126) | No. pregnancies without PPCM  (N=2 075 101) | Risk ratio*  (95% confidence interval) | p-value | No. pregnancies with PPCM  (N=74) | No. pregnancies without PPCM  (N=1 079 519) | Risk ratio†  (95% confidence interval) | p-value |
| Severe preeclampsia | 15 | 13 062 | 21.3 (12.1-37.6) | <0.001 | 9 | 77169294 | 29.7 (14.2, 61.9) | <0.001 |
| Moderate preeclampsia | 19 | 44 848 | 10.2 (6.20-16.9) | <0.001 | 14 | 20 563 | 16.9 (9.35, 30.5) | <0.001 |
| Gestational hypertension | 5 | 18 558 | 5.19 (2.12-12.7) | <0.001 | 3 | 9294 | 7.24 (2.27, 23.1) | <0.001 |
| Normotensive pregnancy | 87 | 1 998 633 | 1 (ref) | - | 48 | 1 041 946 | 1 (ref) | - |

HDP, hypertensive disorders of pregnancy. PPCM, peripartum cardiomyopathy.

* Risk ratios adjusted for parity, maternal age at delivery, multiple pregnancies, and calendar period at delivery.

† Risk ratios adjusted for parity, maternal age at delivery.
